# Carry-Over Effects of Nonbreeding Habitat on Start-to-Finish Spring Migration Performance of a Songbird

**DOI:** 10.1371/journal.pone.0141580

**Published:** 2015-11-03

**Authors:** Emily A. McKinnon, Calandra Q. Stanley, Bridget J. M. Stutchbury

**Affiliations:** Department of Biology, York University, Toronto, Ontario, Canada; Pennsylvania State University, UNITED STATES

## Abstract

For migratory animals, conditions during the nonbreeding period may carry-over to influence spring migration performance. Animals in low-quality habitats are predicted to be in poorer condition, show later migration timing, and travel at slower speeds. This can result in subsequent negative effects on fitness. We tested the hypothesis that nonbreeding season body condition and habitat quality carry-over to affect spring migration performance of a long-distance migratory songbird, the Wood Thrush (*Hylocichla mustelina*). We tracked individual birds between multiple breeding sites in North America and nonbreeding sites in Central America. First, we compared body condition of nonbreeding birds migrating to the same general region of the breeding range with spring migration performance (timing, speed, and duration) obtained from light-level geolocators. Second, we assessed the Normalized Difference Vegetation Index (NDVI) as a proxy for nonbreeding habitat quality, and predicted that birds from wetter habitat or in wetter years (higher NDVI) would show improved migration performance relative to birds from drier sites. We found no evidence of individual-level carry-over effects of nonbreeding season body condition on spring migration performance. Lower NDVI of nonbreeding habitat resulted in delayed spring migration departure, but this effect disappeared by arrival at breeding sites. Birds occupying drier nonbreeding sites migrated faster and for fewer days, compensating for their relatively late departure. We also documented a broader pattern in NDVI and migration timing and distance, in that birds that occupied the wettest areas in the southern part of the nonbreeding range departed significantly later and migrated farther. Our results suggest that individual carry-over effects of nonbreeding habitat quality may be compensated for by a faster and shorter migration strategy. At a broad scale, consistently later spring timing and longer migration distances were associated with the wettest areas (the highest quality habitats) of the Wood Thrush non-breeding range. This supports the theory that high-quality habitats offset the costs of farther migration, resulting in a leap-frog migration pattern.

## Introduction

It has been demonstrated theoretically [1] and with field data [2–4] that events and processes in one part of an animal’s life cycle can carry-over to influence fitness in subsequent parts of the life cycle [5]. In migratory birds, occupancy of low-quality habitat during the nonbreeding season can result in poor physiological condition at spring migration departure [6], which in turn can influence survival to the next year [7]. Spring departure date from tropical nonbreeding sites is often strongly linked to arrival date at breeding sites [8, 9] which is an important predictor of subsequent reproductive success [10]. Therefore carry-over effects that alter migration speed or timing (e.g. date of departure from nonbreeding site, arrival date at breeding site) can have serious consequences for individual fitness and population dynamics [11–13].

One limitation of studies of nonbreeding site carry-over effects is that tracking small birds from start-to-finish on spring migration was not possible until the recent miniaturization of tracking devices [14]. Previous studies have relied on spring migration departure dates [15] or timing of arrival at stopover sites [16, 17] as proxies for overall individual migration performance. Other studies have found links between individual performance at breeding sites and former nonbreeding habitat quality measured indirectly through stable-isotope analysis [18] or remote sensing [3]. There is evidence for strong endogenous control of spring migration timing [19–21], suggesting that carry-over effects might be constrained by endogenous programs. It is also possible that effects of environmental conditions at nonbreeding sites may not be manifested until after migration departure [22]. Therefore it is important to measure spring migration behaviour along the entire journey to assess if carry-over effects from nonbreeding sites occur at any point from departure to arrival at breeding sites.

We tested the hypothesis that nonbreeding habitat quality would carry-over to affect spring migration performance by tracking individual Wood Thrushes (*Hylocichla mustelina* Gmelin, JF, 1789) over their entire spring migration to their breeding sites. Wood Thrushes are a rapidly declining forest songbird that breeds in eastern North America [23]. Threats related to nonbreeding season habitat are not well understood [24], although it is known that the core nonbreeding range for Wood Thrush has undergone extensive deforestation [25]. The month prior to spring departure is the most likely time period to cause carry-over effects on spring migration. Geolocator tracking has shown that Wood Thrushes in Central America typically depart on migration in early April [9]. Prior to departure, birds require sufficient resources to undergo lipogenesis and muscle hypertrophy in preparation for migration [26]. Habitat tends to be at its least productive in Mar-Apr, as it is the peak of the dry season in Central America [27]. Carry-over effects of late nonbreeding season (March) rainfall on spring migration departure date of Neotropic-Nearctic migrant songbirds have been shown in other species [15]. Body condition of Wood Thrushes is variable late in their nonbreeding period (dry season), and declines in parallel with habitat moisture and food availability (both arthropods and fruit) [27], suggesting that carry-over effects on spring migration could occur. Furthermore, climate change models project decreased rainfall and stronger dry seasons in northern Central America [28, 29], underlining the importance of understanding if habitat moisture affects Wood Thrushes via carry-over effects on spring migration performance.

We used three approaches to examine effects of nonbreeding habitat on Wood Thrushes. First, we assumed that birds occupying low-quality nonbreeding territories would exhibit poor body condition compared to birds occupying high-quality territories [30]. Therefore, we tested for effects of body condition of individual Wood Thrushes on their spring migration performance, which we assessed by measuring timing, overall speed, duration (days travelled), and distance (km travelled). We predicted that birds in poorer condition would show later migration departure [6, 15] and subsequent timing over the entire migration route resulting in delayed breeding arrival [2]. We also predicted poor nonbreeding conditions would result in birds spending more days on migration [31], and traveling at a slower speed (total distance/duration). There are several alternative predictions for the effects of nonbreeding conditions on migration distance; birds in poor condition may be physiologically unable to migrate long distances, or they may arrive when breeding territories are already saturated (owing to later timing) or stopovers depleted of food, and be forced to travel further. However, given the expected high physiological costs of migration itself, we predicted that birds in poorer condition would be more likely to opt for shorter duration, even if it resulted in an individual settling in saturated habitat.

Second, we compared habitat quality of nonbreeding sites to migration performance for birds tracked from the same breeding site in Pennsylvania (USA). We measured habitat quality remotely for these individuals by using the Normalized Difference Vegetation Index (NDVI), a satellite-derived index of habitat moisture that correlates with primary productivity and leaf area [32]. Primary productivity is an important predictor of arthropod abundance in tropical forests [33], and thus NDVI can provide an index of overall habitat quality [3]. Wood Thrushes also consume fruit during the nonbreeding season; however, we found that both fruit and arthropod resources declined in abundance in parallel with seasonal moisture [27]. We predicted that individual Wood Thrushes occupying nonbreeding regions with lower NDVI in March (prior to spring migration departure) would show negative carry-over effects on spring migration, such as later departure, slower migration speed, longer duration, shorter migration distance, and later arrival at breeding sites, relative to birds breeding at the same site but occupying wetter nonbreeding habitats.

Finally, we examined carry-over effects by assessing migration timing and performance for two widely separated nonbreeding populations, in Belize and Costa Rica. We predicted that in drier years the average migration performance within populations would be lower (e.g., mean departure date would be later), similar to overall later migration and or breeding phenology observed in other species in dry or drought years [31, 34]. We also predicted that between-population differences in migration performance would be related to differences in NDVI, in that nonbreeding sites that are consistently wetter would support consistently better migration performance of birds from that site. Wood Thrush exhibit a leap-frog migration pattern [25, 35]; therefore, we predicted that birds from Costa Rica should have a longer migration distance than birds from Belize. The longer migration of the Costa Rican Wood Thrushes can be supported if the costs are offset by increased resource availability in wetter forest [36, 37]. If this longer migration distance is supported by higher quality habitat (i.e. higher NDVI), we also expected Costa Rica birds to show overall better migration performance relative to Belize birds (e.g. faster, shorter duration, fewer stopovers). For timing, we expected that selection would favour later migration timing for Costa Rica birds, given that their more northerly breeding sites would be less advanced in phenology than southern breeding sites of Belize birds [38].

Overall, we aimed to provide multiple tests of the hypothesis that nonbreeding processes affect migration patterns of Wood Thrushes, by using direct tracking of birds from multiple breeding and nonbreeding sites. We are also explored how nonbreeding habitat quality may contribute to the leap-frog migration pattern documented in this species. Wood Thrushes are a species of conservation concern in many jurisdictions e.g., [39]). Carry-over effects have potential to influence reproductive success of this species, if nonbreeding habitat results in delays in breeding arrival [34]. Furthermore, climate change is predicted to result in more very dry seasons (< 50% usual rainfall), increased frequency of drought, and drier rainy seasons in Central America [29], and thus, negative carry-over effects of nonbreeding habitat have the potential to be amplified in future. If the leap-frog migration pattern is indeed correlated with broad-scale differences in nonbreeding habitat quality, changes in moisture patterns in Central America could weaken this species-wide pattern, with implications for population dynamics [40, 41].

## Materials and Methods

### Ethics statement

The York University Animal Care Committee approved all protocols for this research. Field permits were obtained from the Belize Forest Department and MINAE in Costa Rica. Permission to use bird bands on migratory species in Central America was obtained from the Bird Banding Office in Canada.

### Field methods

Wood Thrushes were captured for geolocator-tagging at the Belize Foundation for Research and Environmental Education (BFREE, 16.5°N, -88.7°W, www.bfreebz.org) in the Toledo district of Belize, Central America, over the course of 4 nonbreeding seasons, from 2010 to 2013 (n = 166). For our range-wide sample, birds were also captured at Hemlock Hill Biological Station (41.8°N, -79.9°W), in Pennsylvania, USA, during the 2008–2011 breeding seasons (n = 96) and tracked to multiple nonbreeding sites, and at La Selva Biological Station (10.4°N, -84.0°W) in Costa Rica over three nonbreeding seasons, from 2009 to 2012 (n = 109) and tracked to multiple breeding sites. Geolocators (British Antarctic Survey model MK14S, 1.6g, 10–15mm stalk length) were deployed on birds by using a Teflon ribbon leg-loop harness, custom fit to each bird. We attempted to recapture all birds with geolocators one year later, at deployment locations. Once recaptured, we removed the geolocators and released the birds.

Individual birds were also marked with metal and plastic leg bands for identification in the field and upon recapture. Birds captured outside of the breeding season were genetically sexed by polymerase chain reaction (PCR) amplification of sex-linked genes [42] using a small blood or feather sample see detailed methods in [43]), and birds were aged as juvenile (first-year) or older by examination of plumage characteristics [44].

### Geolocator analysis

Geolocator data were downloaded and analysed using the light-threshold method, following methods by McKinnon *et al*. [45]. We extracted 6 spring migration variables from the light data for further analyses: last noon at nonbreeding site (‘departure date’), first noon north of the Gulf of Mexico (‘cross date’), first noon at breeding site (‘arrival date’), total migration distance (km), total migration duration (days), and overall migration speed (km/d). To calculate migration distance, we used a straight-line measure starting at nonbreeding sites and connecting stopovers (i.e. ≥ 2 noon locations differing by less than 2° in longitude), until arrival at breeding sites. Arrival (and departure) dates were determined by examining locations to assess when longitudes varied by < 2°, latitudes were within the known breeding or wintering range, and location remained consistent for the duration of the recorded breeding or wintering period. When we had data available for periods when birds were known to be at breeding or wintering sites, we used the standard deviation in latitude and longitude to assess the typical variation at a stationary point and used this standard deviation to help define arrival and departure dates (i.e. locations shifting 2° or more than the expected variation at a given stationary site were considered to have left that site). For birds captured at our breeding study site (Pennsylvania), we calculated the location of nonbreeding home ranges by taking the average latitude and longitudes for January and February, using a nonbreeding-site calibrated sun elevation [45]. For birds captured at both nonbreeding study sites (Costa Rica and Belize), we used a similar approach to calculate the average latitude and longitude for June and July, using a breeding-site calibrated sun elevation [45].

### Body condition (Belize site only)

For birds captured during the dry season in Belize, we measured the metatarsus bone to the nearest 0.1mm, and weighed each bird to the nearest 0.1g. We also scored fat levels on a scale from 0–7, following established protocols for monitoring nonbreeding migratory birds [46]. Following methods of other studies on nonbreeding migrants e. g., [30, 47, 48]) we determined body condition by calculating the estimated lean mass of each individual using an equation derived from a regression of fat-free bird weight versus tarsus. We then calculated each bird’s predicted lean mass based on its tarsus length, and compared the actual weight of the bird to the predicted lean mass. The difference was converted to a percentage for easier interpretation, i.e. a condition index of 8.6% indicates a bird is 8.6% larger than its expected lean mass.

Since we captured each individual bird on a different day relative to its departure date (on average, 55 days before departure, range of 24–95 days), we adjusted the body condition of each to account for difference in capture date. Previous work [27] found that Wood Thrush body condition (% predicted lean body mass) at our study site declined significantly over the nonbreeding, at a rate of 0.04% per day (95% confidence intervals: 0.02–0.05). Therefore we multiplied the number of days between capture and departure date by -0.04% to estimate departure body condition for each bird. This resulted in a decrease in the lean mass of 2.2%, on average (range of 0.96–3.64%), from the date of capture to the known departure date.

### Normalized Difference Vegetation Index analyses

To determine an estimate of habitat moisture for different nonbreeding regions, we used the Normalized Difference Vegetation Index (NDVI). This commonly-used ecological index is derived from satellite imagery, and higher values indicate increased leaf area and primary productivity [32]. Central American NDVI data for March from 2009–2013 were downloaded from the Land Processes Distributed Active Archive Center (lpdaac@usgs.gov), and clipped to the Wood Thrush nonbreeding range (natureserve.org) by using ArcGIS 10 (ESRI). In ArcGIS, we created a 100-km radius buffer around the estimated nonbreeding home range of each Wood Thrush from our PA breeding site. This accounts for most error in geolocator position for birds tracked from breeding to nonbreeding sites [45]. Within this 100-km radius circle we extracted all NDVI values, and calculated the average NDVI. For our two Central American study sites, we used a 100-km buffer centred on each field station (BFREE in Belize, and La Selva Biological Station in Costa Rica) for a comparable NDVI average. We used March NDVI values for all, as this is the last month that Wood Thrushes were resident at their nonbreeding sites before departing on spring migration and previous work has found that rainfall in March predicts departure dates in another Neotropical migrant [15].

### Statistical analyses

Previous studies on Wood Thrushes have documented no effect of geolocators on return rates [25] or short-term (i.e. within-nonbreeding season) effects on body condition [49]. To assess if birds that survived to return to our study sites with geolocators were larger than average (i.e. if geolocators disproportionately affected survival of smaller birds, for example) we compared the pre-migration body condition of returning birds in Belize with that of all birds captured during the dry season in Belize by using a t-test (both groups were normally distributed according to a Shapiro-Wilk normality test; W = 0.99, P = 0.20 and W = 0.96, P = 0.43 for dry-season birds and returning geolocator birds, respectively).

To determine if late season Wood Thrush body condition in Belize predicted subsequent migration performance, we used general linear mixed models (GLMMs) with spring migration variables as responses and Belize Wood Thrush departure condition as our predictor of interest. Since both age and sex have strong effects on migration timing and stopover behaviour [49], we also included these as independent fixed effects. We included year of migration and the identity of the individual bird as random effects.

To quantify patterns in NDVI for the Wood Thrush nonbreeding range overall, we used simple linear models with NDVI of all Wood Thrush nonbreeding sites as the response and latitude and longitude as predictors. For comparison of NDVI values to migration performance of birds, we conducted separate analyses for breeding site versus nonbreeding site deployments. For breeding site birds (Pennsylvania USA deployments), we used GLMMs with spring migration variables as responses, March NDVI and sex as fixed effects, and individual as a random effect. We used the March NDVI from the specific year in which each bird was tracked, therefore we did not include year as a separate factor. We did not include age in this analysis since all birds were adults undergoing spring migration for at least their second time. For nonbreeding site birds (Belize and Costa Rica deployments), we used a similar approach but we nested March NDVI by study site, to examine if yearly variation in NDVI within each site was associated with effects on population-level migration performance. We also ran the model without nesting to determine if across sites higher NDVI was related to better spring migration performance. All statistical analyses were performed by using program R [50] and means are reported with standard error unless otherwise stated. For mixed effects models, we used the package lmerTest [51] to generate P-values and degrees of freedom for fixed effects by using Satterthwaite’s approximations.

## Results

We recaptured 86 Wood Thrushes wearing geolocators. Some geolocators failed to record any usable data, therefore our final sample size was: n = 26 (Belize, including 3 birds tracked twice), n = 21 (Costa Rica, including 4 birds tracked twice), and n = 23 (Pennsylvania including 5 birds tracked twice). Since it is only possible to obtain migration information from birds that survive to return to study sites, this may have biased our results to individuals in better body condition and perhaps better able to carry geolocators. We tested for this by calculating an estimated condition index for all birds captured in Belize during the dry season (n = 190), and compared with the condition of birds that returned with geolocators. The difference in condition was not significant (*t*-test, *t* = -0.37, df = 32.1, P = 0.71); birds that subsequently returned wearing geolocators were on average 3.8% above their expected lean body mass during the dry season prior to spring migration, while birds that did not return with geolocators had a condition index of 3.2%.

**Fig 1:**
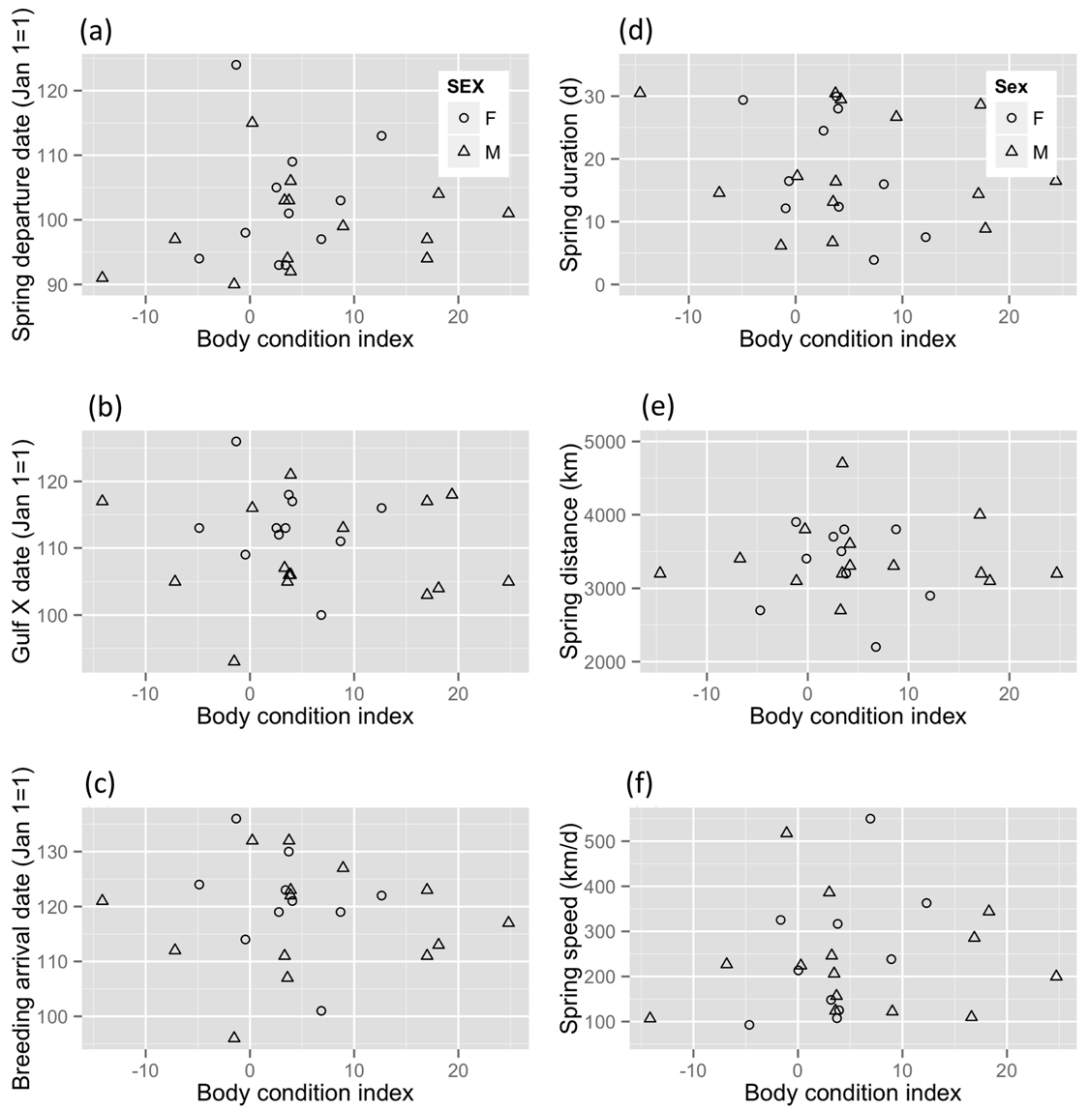
Estimated body condition of Wood Thrushes at a nonbreeding site in Belize and its relationship to migration performance. (a) Spring migration departure date (n = 26), (b) date individuals crossed the Gulf of Mexico (n = 26), (c) date individuals arrived at their breeding sites (n = 24), (d) spring migration duration (# of days spent migrating) (n = 24), (e) spring migration distance (km travelled), and (f) spring migration speed (km/d) (n = 24).

Estimated departure body condition of Wood Thrushes in Belize ranged widely from -15% lean body mass to 23.3% lean body mass. Body condition in Belize was not a significant predictor of an individual bird’s spring migration timing at any stage (Table A in S1 File) when controlling for known age- and sex-effects, and random variation by year and among individuals (Fig 1A–1C). Likewise, birds in better body condition were not more likely to migrate faster or for fewer days (Table A in S1 File, Fig 1E and 1F).

**Fig 2:**
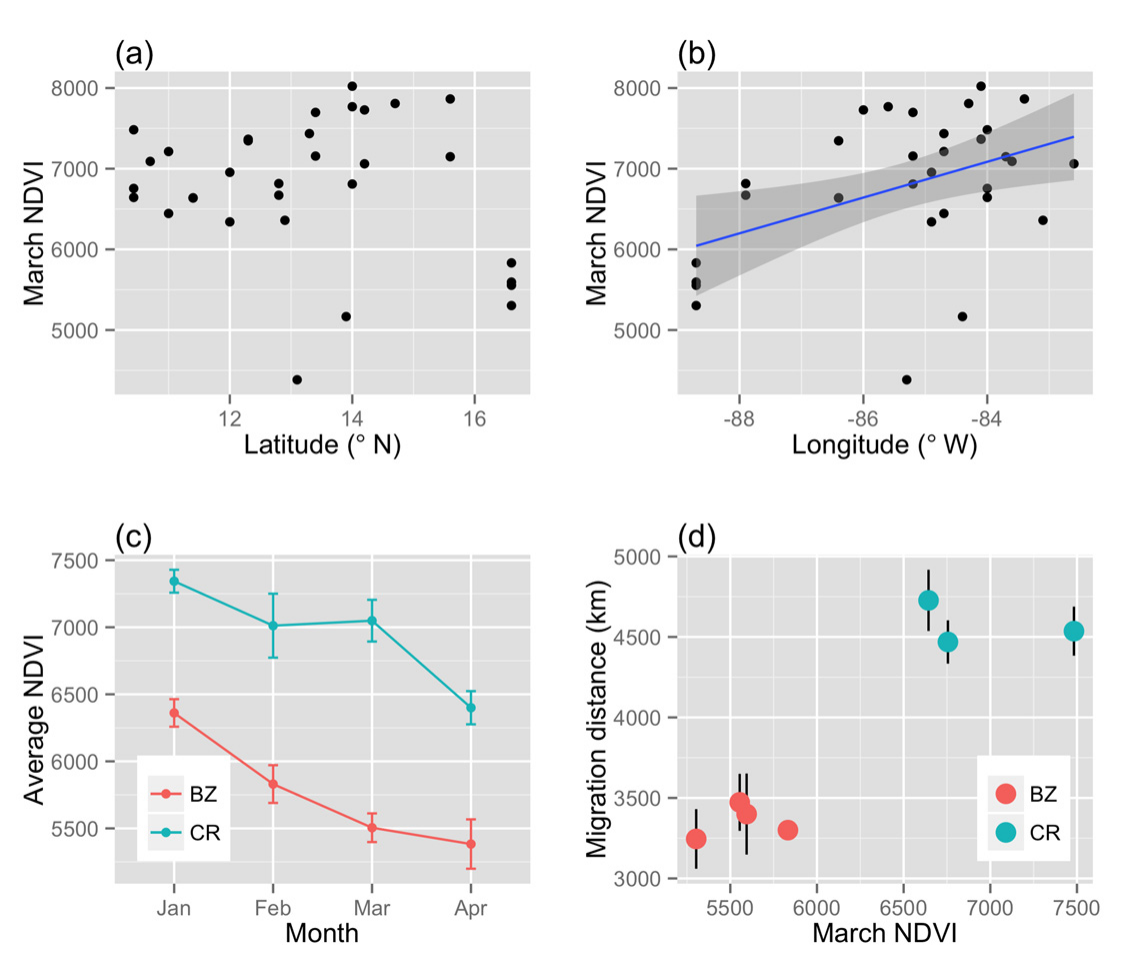
Wood Thrush nonbreeding range patterns in late season (March) NDVI. Average March NDVI across (a) latitudes, and (b) longitudes. (c) NDVI values throughout the nonbreeding season at study sites in Belize and Costa Rica. (d) Average migration distance (with vertical standard error bars) and March NDVI (only 1 bird tracked in Belize in 2010; therefore no error bars are shown for this point).

NDVI values varied across the nonbreeding range with consistently wetter sites in the east of the nonbreeding range, and drier sites in the west (r^2^ = 0.17, F_1,30_ = 7.50, P = 0.01) (Fig 2B). There was a trend for sites in the north of the nonbreeding range to be drier as well (estimate of -100.07 ± 80.81 NDVI for each degree of latitude), but latitude alone was not a significant predictor of nonbreeding site NDVI (r^2^ = 0.02, F_1,30_ = 1.53, P = 0.22) (Fig 2A). The Costa Rica site was consistently wetter than that in Belize during the course of our study (2009–2013) (Fig 2C) over the period from January to April, when most Wood Thrushes are resident. NDVI also declined from mid- to late season at both sites. NDVI in March correlated significantly with population-level migration distance of Wood Thrushes tracked from Belize versus Costa Rica (Fig 2D) in that birds from the wetter site, Costa Rica, migrated on average >1000km farther than birds from Belize (mixed effects model estimate for the effect of March NDVI on migration distance: 0.69 ± 0.12, t = 5.66, df = 10.81, P < 0.001).

**Fig 3:**
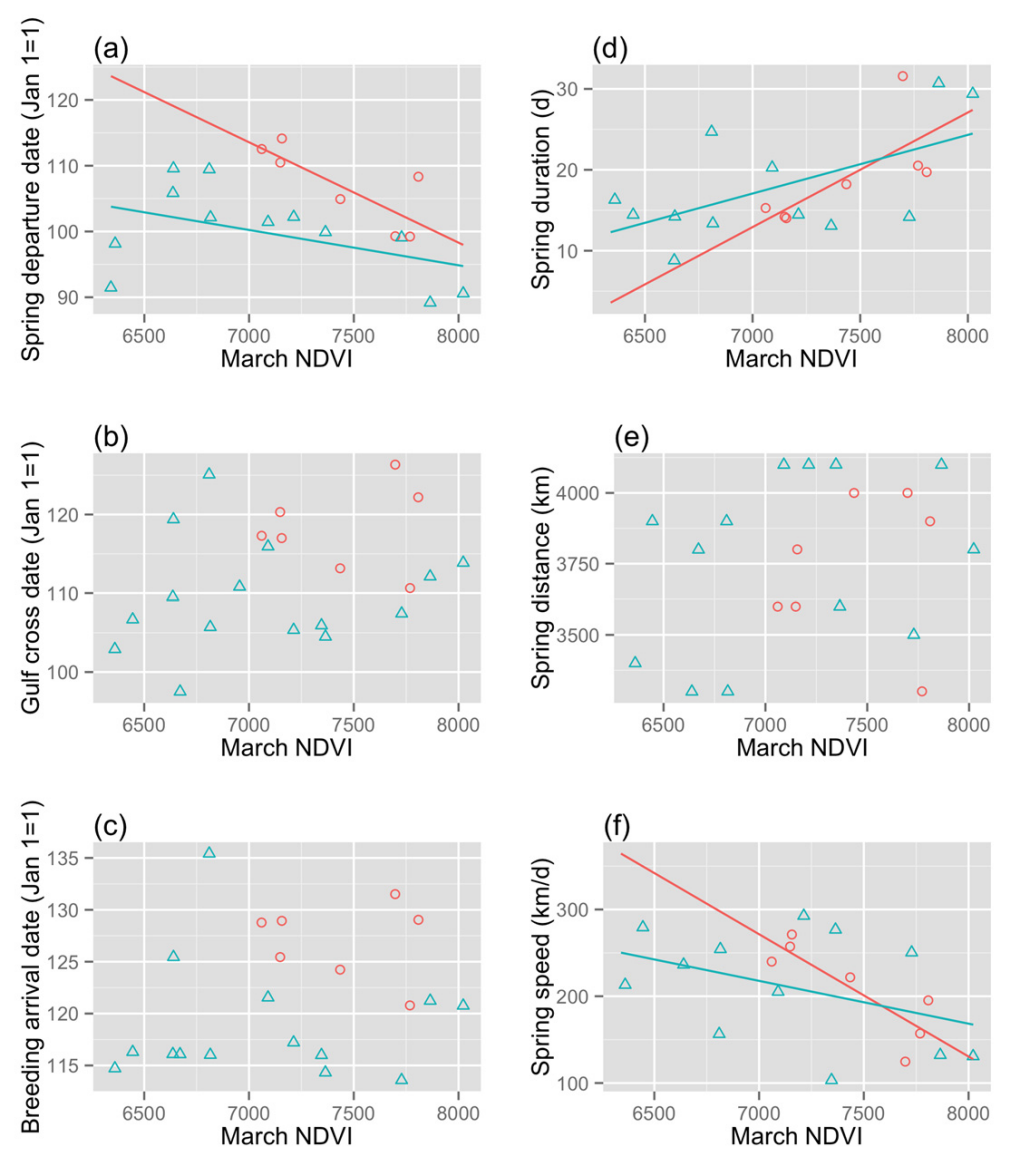
Nonbreeding site NDVI and spring migration performance for Wood Thrushes that bred at the same site in Pennsylvania, USA. Migration measures include: (a) date of departure from nonbreeding site (n = 21), (b) date crossing the Gulf of Mexico (n = 24), (c) date of arrival at breeding sites (n = 23), (d) spring migration duration (# of days on migration) (n = 21), (e) spring migration distance (km travelled), and (f) spring migration rate (km/d) (n = 21).

Breeding birds tracked from Pennsylvania migrated to the central part of the Wood Thrush range (Fig A in S1 File) [25], to sites with an average ± SE NDVI of 6957 ± 165, and range of 4384–8022, depending on the year and the estimated location of the bird. NDVI values for two individuals were very low and statistical outliers (>1.5 times the interquartile range); therefore, we removed these individuals from model analyses. NDVI in March was a significant predictor of an individual’s migration timing at departure, in that birds that occupied wetter nonbreeding habitat departed earlier (Fig 3A, Table B in S1 File). However, the relationship between NDVI and migration timing was no longer apparent mid-migration at the Gulf of Mexico, or at arrival at breeding sites (Fig 3B and 3C). NDVI in March was a significant predictor of overall spring migration duration (Fig 3D, Table B in S1 File), in that birds occupying wetter nonbreeding habitat spent longer on migration. NDVI in Mar was not a predictor of migration distance within the Pennsylvania-breeding birds (Fig 3E), but overall migration speed (km/d) was significantly related to nonbreeding NDVI (Fig 3F). In this case, birds occupying wetter nonbreeding habitat were slower overall (Table B in S1 File).

**Fig 4:**
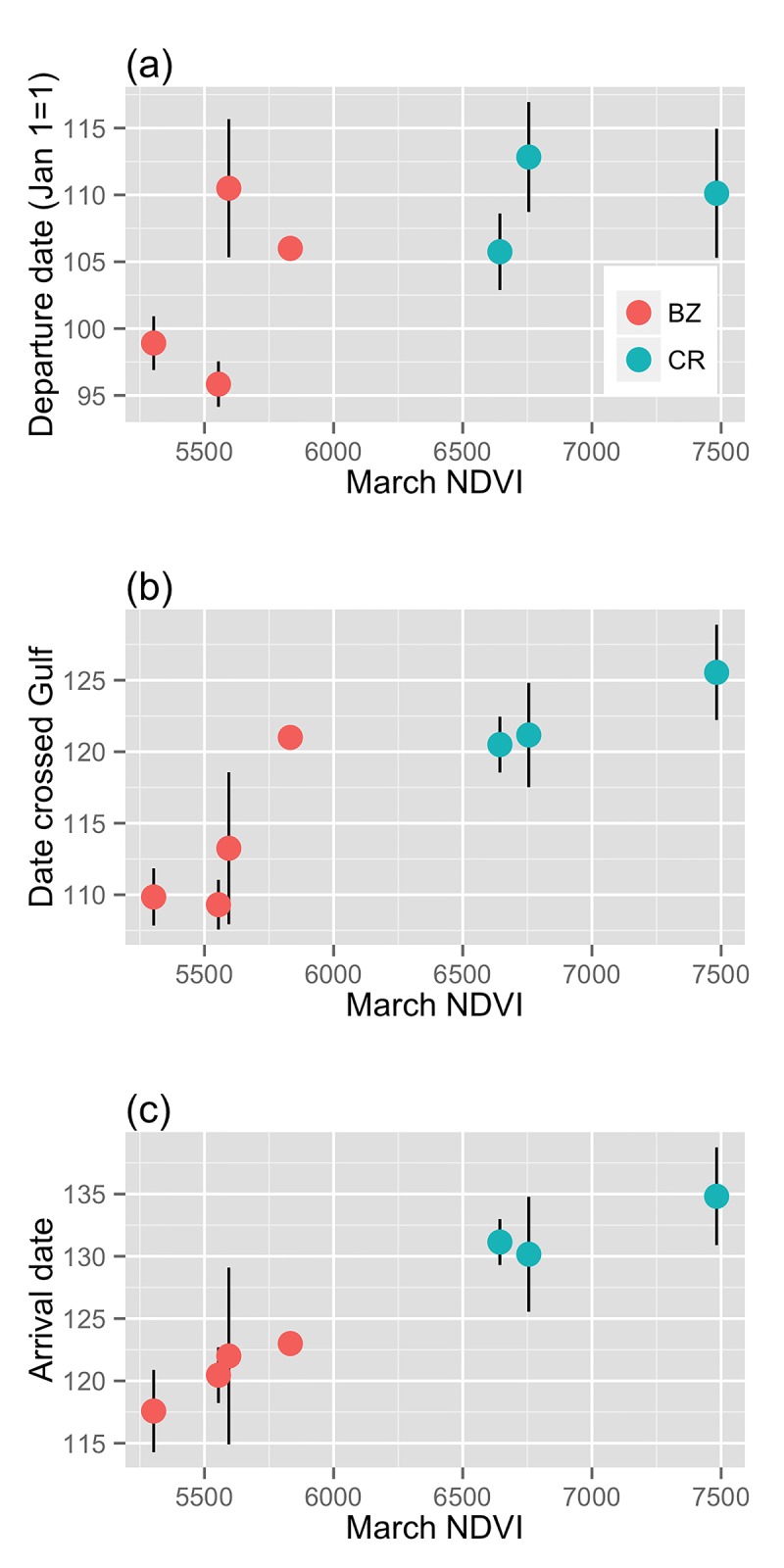
March NDVI and spring migration timing of Wood Thrushes occupying nonbreeding sites in Costa Rica (CR) and Belize (BZ). Timing was measured at three points: (a) departure from nonbreeding sites, (b) date crossing the Gulf of Mexico, and (c), and arrived at their breeding sites later. Points show means from tracked birds each year with standard error (except for one year in Belize with only 1 individual tracked and no error bars).

**Fig 5:**
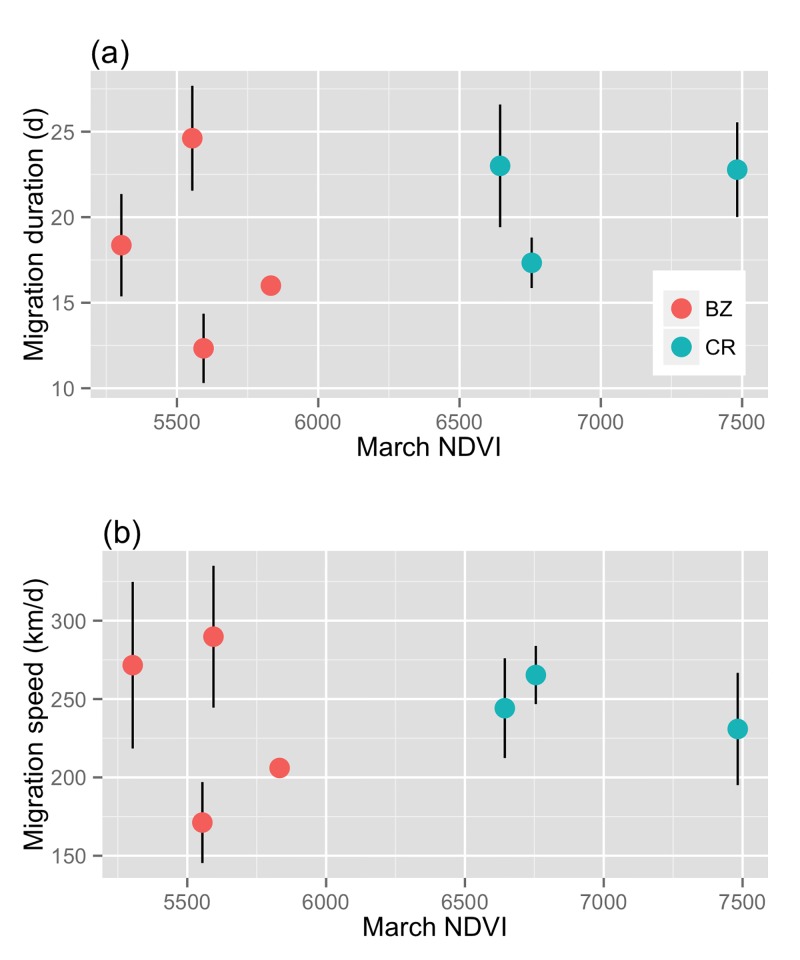
March NDVI and average migration duration (a) and migration speed (b) for Wood Thrushes tracked from Belize and Costa Rica. Points show mean values for each year by site with vertical standard error bars.

For Belize and Costa Rica birds, inter-annual differences in NDVI were not related to differences in within-population spring migration performance (Table B in S1 File). However, NDVI in March was a significant predictor of migration timing differences between populations (Fig 4) in all mixed-effects models. Likelihood ratio tests of models for NDVI and migration timing of birds from Belize and Costa Rica without the random effects of year and individual identity indicated no significant difference from a simple linear model. Therefore we used a simple linear model, and found that March NDVI was a significant predictor of spring migration departure date: birds departed on migration later when occupying wetter habitat at the Costa Rican site (r^2^ = 0.32, F_3,48_ = 8.9, P <0.001). This relationship was even stronger at the crossing of the Gulf of Mexico and at arrival to breeding sites (gulf cross: r^2^ = 0.60, F_3,52_ = 28.02, P <0.001; arrival date: r^2^ = 0.57, F_3,49_ = 24.3, P = <0.001). March NDVI was not a predictor of spring migration duration or speed, either within population (Table B in S1 File) or between populations (Fig 5) (mixed effects model estimate for duration: -0.003 ± 0.002, t = -0.17, df = 20.09, P = 0.86; speed: 0.01 ± 0.02, t = 0.46, df = 19.28, P = 0.65).

## Discussion

We did not detect any carry-over effects of pre-migration body condition on individual spring migration performance of a Neotropical migratory songbird (Fig 1). However, variation in nonbreeding habitat NDVI (indicative of moisture and correlated with food abundance) was a predictor of spring departure date, spring migration duration, and overall speed for birds breeding in the same forest patch in Pennsylvania, USA (Fig 3). Annual differences in NDVI at two nonbreeding sites in Belize and Costa Rica did not correlate with the mean spring migration performance of birds from those sites, i.e. wetter years did not result in better migration performance from birds at that site, on average. We documented a broader-scale pattern in that the Costa Rica Wood Thrushes, which occupied consistently wetter tropical habitat, migrated farther distances in spring (Fig 2D) and showed later migration timing (Fig 4), relative to the Belize population. The earlier phenology of breeding sites for Belize birds combined with an increasingly hostile (dry) nonbreeding habitat likely drives their earlier spring migration timing relative to Costa Rica birds, which breed further north and occupy higher quality winter habitat.

Broad variation in body condition of nonbreeding birds in Belize did not correlate with their individual migration performance, in contrast to our predictions that poor body condition would cause negative carry-over effects on migration performance. In American Redstarts (*Setophaga ruticilla*), another small Neotropical migratory songbird, a decline in body condition of 8% over the nonbreeding season resulted in a 6-day delay in spring migration departure [52]. A recent study of Black-and-White Warblers (*Mniotilta varia*) at a migration stopover found a more complex relationship between body condition and migration performance; birds arriving first (i.e. better migration performance) were in poorer condition than those arriving later, but birds arriving during the mid-migration period were in the best body condition [17]. Thus body condition may be related to overall migration strategy. For example, Wood Thrushes from Belize may use a strategy that entails fuelling up at stopovers in the northern Yucatan peninsula or elsewhere, and compensating *en route* for any differences in departure condition. It is also possible that Wood Thrushes from Belize, which migrate relatively short distances (~3300km on average, range 2200–4800km) to breed in south-eastern U. S. may not require high levels of fat or muscle required for longer-distance migrations. Regardless, carry-over effects of nonbreeding body condition on individual migration performance were not apparent.

We found some support for our predictions when assessing NDVI of nonbreeding habitat for Wood Thrushes from the same breeding site. NDVI was correlated with migration departure, duration, and rate, but not other measures of timing or distance. That the correlation with migration timing was not apparent beyond departure dates (and critically, not at breeding arrival), suggests that birds may be able to compensate for delays caused by poor-quality habitat. In fact, the birds occupying relatively dry sites left later, but they also spent less time on migration and travelled more quickly, indicating that they can compensate for a later start. Further studies of start-to-finish migration strategies could elucidate whether an early-but-longer migration has greater fitness benefits than the late-start, faster migration strategy.

We also found a significant relationship between NDVI and migration performance when comparing birds from Belize and Costa Rica. These two study sites were consistently different in terms of habitat moisture (Belize always drier) and in migration strategy (Belize birds migrated shorter distances, and migrated earlier). This broad-scale relationship likely contributes to the range-wide parallel, leap-frog migration system documented in this species [25, 35], where birds occupying nonbreeding sites further southeast migrate farthest northeast to breed. The disadvantage of occupying a relatively dry region (e.g. Belize) could be compensated for by the fact that these populations can start breeding earlier. Staying longer at relatively productive nonbreeding sites, e.g. Costa Rica, would allow for greater pre-migratory fattening that could reduce the number of spring stopovers [53]. Indeed, despite a longer migration on average, Costa Rica Wood Thrushes stopped for about the same number of nights as Belize Wood Thrushes (McKinnon, Stanley, and Stutchbury, unpublished data). Wood Thrushes occupying nonbreeding sites further south also have longer wings [35], indicating that these populations have evolved morphological adaptations for longer migratory flights [54].

The mechanisms driving the evolution of leap-frog migration, a pattern that is widespread within and among species, are still not well understood [55]. In the case of Wood Thrushes, asymmetric competition would not appear to drive the pattern, as birds occupying nonbreeding habitat in the north of the range (Belize) are smaller than those in the south (Costa Rica) [35]. Furthermore, Wood Thrushes from Costa Rica and Belize tracked for our study arrived to the Tropics in fall at approximately the same time (median date crossing into the Tropics, Belize Wood Thrushes: 16 Oct, range 9–28 Oct; Costa Rica Wood Thrushes: 18 Oct, range 2 Oct—1 Nov), giving neither group a competitive advantage at northern nonbreeding sites in terms of timing (Fig B in S1 File). Instead we suggest the observed gradient in nonbreeding habitat quality (Fig 2), combined with differences in breeding phenology and thus migration timing (Fig 4), are the drivers of the leap-frog migration pattern in this species, in that higher quality habitats in the south of the nonbreeding range support longer migrations, while relatively low-quality habitats constrain birds to leave earlier and migrate shorter distances [36, 37].

Several studies have shown that birds in the same breeding population that arrive from more wet nonbreeding habitats (inferred from stable isotope analysis) produce more young [2, 3, 18], an effect mediated by earlier migration timing [17], such that they arrive at breeding sites earlier. However, a study using stable isotopes to infer migratory connections of an Arctic-breeding population of Yellow Warblers (*Setophaga petechia*) did not detect any carry-over effects of nonbreeding habitat on arrival date or reproductive success [56],. Carry-over effects of nonbreeding habitat moisture (assessed by stable isotope analysis) were also undetected in Magnolia Warblers (*Setophaga magnolia*) captured during spring migration [57]. Recent year-round tracking of a long-distance migratory shorebird did not detect any individual-level carry-over effects of delayed spring migration on survival or breeding success and suggested that this may be a result of strong selection on endogenous programs for optimal timing of arrival at breeding sites [58]. All of these results suggest that individual-level carry-over effects may vary in strength (or detectability) by species, based on distribution, habitat requirements, or variation in the strength of endogenous control of migration.

Overall our results show that carry-over effects of nonbreeding body condition are absent in our study population, and that nonbreeding habitat moisture has some effects on migration strategy (wetter nonbreeding territories associated with earlier departure, longer duration and slower speed for birds from the same breeding population) but does not affect breeding arrival date. Our data also suggest that the species-level parallel, leap-frog migration pattern in Wood Thrushes could be driven by a range-wide gradient in nonbreeding habitat quality combined with differences in breeding site phenology. This pattern results in populations occupying nonbreeding habitat further southeast in more productive habitat migrating further and later than populations occupying drier habitat to the north. This hypothesis could be further explored in other species that show a gradient in nonbreeding habitat quality over a broad geographical area that would produce migration distance differences.

## Supporting Information

S1 File General linear mixed effects model results for tests of body condition as a predictor of spring migration performance of individual Wood Thrushes from a study site in Belize (Table A). Results of mixed effects linear models of the effect of March NDVI on individual migration performance for Wood Thrushes from the same breeding site (PA = Pennsylvania) and population-level migration performance at two nonbreeding sites (BZ = Belize, CR = Costa Rica) (Table B). Migratory origins and destinations of birds breeding in Pennsylvania and from nonbreeding sites in Belize and Costa Rica (Figure A). Histogram of arrival dates to the Tropics (first date south of 24.5°N) for Wood Thrushes that eventually occupied nonbreeding sites in Belize and Costa Rica (Figure B).
(DOCX)
