# Supplementary material for: Carry-Over Effects of Nonbreeding Habitat on Start-to-Finish Spring Migration Performance of a Songbird
Source: PLoS One. 2015 Nov 3;10(11):e0141580. doi: 10.1371/journal.pone.0141580 (PMC4631350; doi:10.1371/journal.pone.0141580)
Supplement: S1 File — Results of mixed effects linear models of the effect of March NDVI on individual migration performance for Wood Thrushes from the same breeding site (PA = Pennsylvania) and population-level migration performance at two nonbreeding sites (BZ = Belize, CR = Costa Rica) (Table B). Migratory origins and destinations of birds breeding in Pennsylvania and from nonbreeding sites in Belize and Costa Rica (Figure A). Histogram of arrival dates to the Tropics (first date south of 24.5°N) for Wood Thrushes that eventually occupied nonbreeding sites in Belize and Costa Rica (Figure B). (DOCX) [file pone.0141580.s001.docx]

S1 File Supplemental Materials - Carry-over effects of nonbreeding habitat on start-to-finish spring migration performance of a songbird

McKinnon, E.A., Stanley, C. Q., Stutchbury, B.J. M.

Table S1. General linear mixed effects model results for tests of body condition as a predictor of spring migration performance of individual Wood Thrushes from a study site in Belize.

Fixed effects of sex and age, and random effects of individual and year were included in all full models, p-values were calculated by Satterthwaite’s approximations.

| Fixed effect | Body condition | | | | |
| --- | --- | --- | --- | --- | --- |
|  | N | Coefficient (SE) | t | d.f. | P |
| Departure date | 26 | 0.08 (0.19) | 0.40 | 21.46 | 0.69 |
| Date crossing Gulf | 26 | -0.11 (0.17) | -0.65 | 21.48 | 0.52 |
| Arrival at breeding | 24 | -0.11 (0.22) | -0.50 | 19.75 | 0.62 |
| Spring duration | 24 | -0.25 (0.21) | -1.17 | 17.62 | 0.56 |
| Spring distance | 24 | -0.02 (0.10) | -0.19 | 19.75 | 0.85 |
| Spring speed | 24 | 1.80 (3.19) | 0.56 | 19.99 | 0.58 |

Table S2. Results of mixed effects linear models of the effect of March NDVI on individual migration performance for Wood Thrushes from the same breeding site (PA = Pennsylvania) and migration performance at two nonbreeding sites (BZ = Belize, CR = Costa Rica). Full models included year and individual as random effects, and sex and age (nonbreeding deployments only) as additional fixed effects. P-values were calculated by Satterthwaite’s approximations.

| Fixed effect | March NDVI | | | | |
| --- | --- | --- | --- | --- | --- |
|  | N | Coefficient (SE) | t | d.f. | P |
| Individual variation within a breeding population (PA) | | | | | |
| Departure date | 21 | -0.007 (0.003) | -2.41 | 16.00 | 0.03 |
| Date crossing Gulf | 24 | 0.001 (0.003) | 0.50 | 18.96 | 0.62 |
| Arrival at breeding | 23 | 0.0002 (0.003) | -0.10 | 17.98 | 0.92 |
| Spring duration | 21 | 0.008 (0.003) | 3.00 | 16.00 | 0.008 |
| Spring distance | 22 | 0.18 (0.14) | 1.30 | 16.94 | 0.21 |
| Spring speed | 21 | -0.06 (0.03) | -2.30 | 16.00 | 0.03 |
| Individual variation within two nonbreeding populations (BZ and CR) | | | | | |
| Departure date | 32  25 | BZ 0.005 (0.009)  CR 0.006 (0.007) | 0.60  0.79 | 1.76  1.74 | 0.61  0.52 |
| Date crossing Gulf | 56 | BZ 0.006 (0.004)  CR 0.006 (0.003) | 1.45  1.93 | 2.62  2.45 | 0.26  0.17 |
| Arrival at breeding | 53 | BZ 0.006 (0.005)  CR 0.006 (0.004) | 1.25  1.56 | 3.45  3.27 | 0.29  0.21 |
| Spring duration | 50 | BZ 0.003 (0.01)  CR 0.002 (0.008) | 0.30  2.78 | 2.79  2.76 | 0.78  0.80 |
| Spring distance | 49 | BZ -0.10 (0.40)  CR 0.08 (0.31) | -0.26  0.27 | 2.89  2.72 | 0.81  0.80 |
| Spring speed | 46 | BZ -0.09 (0.11)  CR -0.07 (0.09) | -0.81  -0.76 | 2.94  2.88 | 0.48  0.50 |


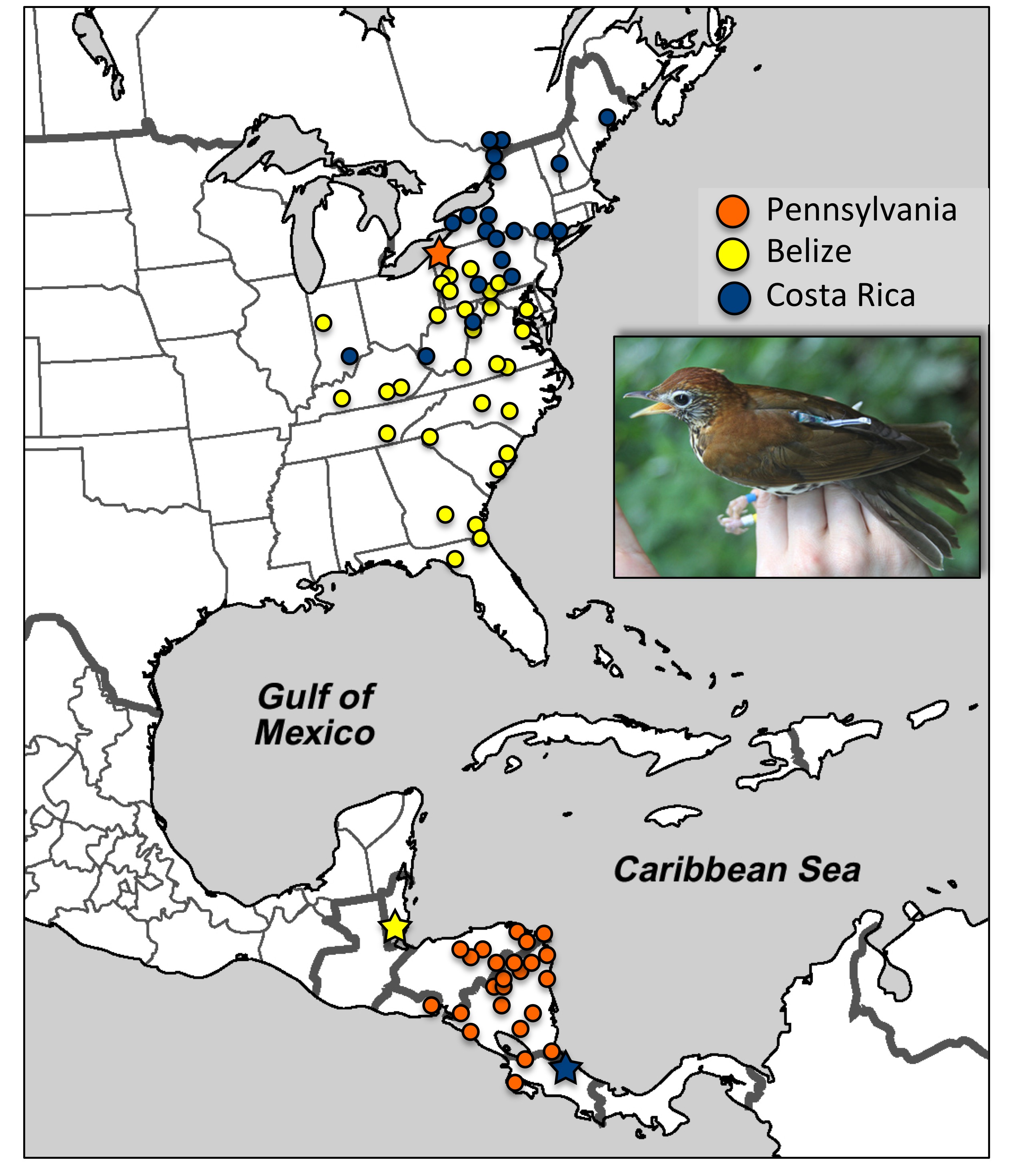


**Fig S1. Migratory origins and destinations of birds breeding in Pennsylvania and with nonbreeding sites in Belize and Costa Rica.**

Deployment sites are indicated by stars, colour-coded circles indicate estimated breeding or nonbreeding destinations for birds from that deployment site. Inset photo shows a Wood Thrush wearing a geolocator (photo credit Kevin C. Fraser). Background map shows state and provincial boundaries for North American countries by thin grey lines and country divisions by thick grey lines.


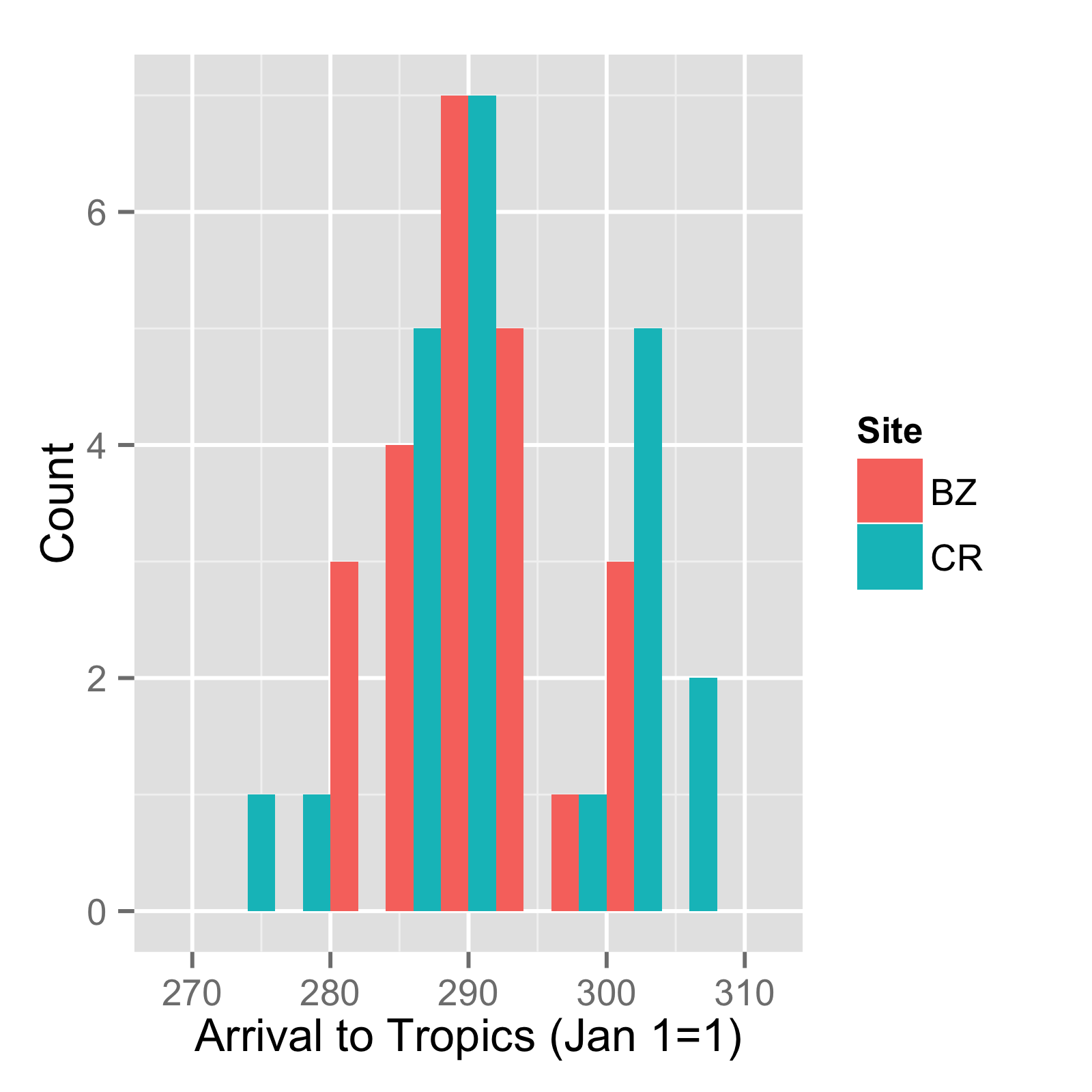


**Fig S2. Histogram of arrival dates to the Tropics (first date south of 24.5°N) for Wood Thrushes that eventually occupied nonbreeding sites in Belize and Costa Rica.**
